# Myostatin genotype regulates muscle-specific miRNA expression in mouse pectoralis muscle

**DOI:** 10.1186/1756-0500-3-297

**Published:** 2010-11-11

**Authors:** Satyanarayana Rachagani, Ye Cheng, James M Reecy

**Affiliations:** 1 Department of Animal Science, Iowa State University, Ames, Iowa, USA

## Abstract

**Background:**

Loss of functional Myostatin results in a dramatic increase in skeletal muscle mass. It is unknown what role miRNAs play in Myostatin mediated repression of skeletal muscle mass. We hypothesized that Myostatin genotype would be associated with the differential expression of miRNAs in skeletal muscle.

**Findings:**

Loss of functional Myostatin resulted in a significant increase (p < .001) in miR-1, miR-133a, miR-133b, and miR-206 expression. In contrast, Myostatin genotype had no effect (P > .2) on miR-24 expression level. Myostatin genotype did not affect the expression level of MyoD or Myogenin (P > 0.5).

**Conclusions:**

Myostatin may regulates the expression of miRNAs such as *miR-133a*, *miR-133b*, *miR-1*, and *miR-206 *in skeletal muscle as it has been observed that the expression of those miRNAs are significantly higher in myostatin null mice compared to wild type and heterozygous mice. In contrast, expression of myogenic factors such as MyoD or Myogenin has not been affected by myostatin in the muscle tissue.

## Background

miRNAs have generated strong interest among researchers in different fields to understand their biosynthesis, mechanism of action, and identification of their targets. First discovered in the caenorhabditis elegans as small temporal RNAs required for proper developmental timing, miRNA are a family of highly conserved, non-coding, 22 nucleotide [1] double-stranded RNA products that post-transcriptionally regulate gene action[1-4].

Sampere *et al*. (2004) first identified the expression of the muscle specific miRNas such as miR-1, -133a and -206[5]. Their expression was highly enriched in both human and mouse heart and skeletal muscle. Subsequently, several miRNA expression profiling studies have consistently shown *miR-1*, *-133a *and *-206 *to be muscle specific[6-11]. These muscle specific expressions are evolutionarily conserved among animals[12]. Over expression of *miR-1 *and *miR-133 *during the in-vitro development of embryoid bodies from mouse embryonic stem cells demonstrated that distinct steps in muscle development are specified by cooperative and opposing interactions between *miR-1 *and *miR-133*.

Given that miRNAs have been shown to play roles in the regulation of satellite cell proliferation and differentiation, the current study was carried out to evaluate the effect of loss of Myostatin function on skeletal muscle miRNA expression.

## Methods

### Animal Experimentation

The present experiment was conducted with mice that were MSTN^-/- ^[13], MSTN^+/+ ^and MSTN^+/- ^(either sex). MSTN^+/- ^mice were mated to produce MSTN^-/- ^(n = 9), MSTN^+/+ ^(n = 10) and MSTN^+/- ^(n = 10) animals. The mice were housed in a temperature- and humidity-controlled room and maintained at 22°C on a 12-h light-dark cycle with food and water provided ad libitum. At 1 week of age, mice were individually identified and genotyped. Mice were weaned at 21 days of age. At 35 days of age mice were euthanized by CO_2 _asphyxiation. The pectoralis muscles were excised, flash-frozen in liquid nitrogen, and stored at -80°C until RNA isolation. The Iowa State University Institutional Animal Care and Use Committee approved all procedures that involved mice.

### DNA Isolation and Genotyping

The DNA was isolated by standard protocol with few modifications[14] from toes clipped at seven days after birth. Myostatin genotyping was completed with a three-primer PCR reaction. The primer sequences were: Mstn1-5'-GGC ATC TGT TCT GCT ATT ACG TGC-3', Mstn2-5'-GTG CGA TAA TCC AGT CCC AT-3' and Mstn3-5'-GTG GAT GTG GAA TGT GTG CGA GG-3'. The PCR amplification reaction contained 50 ng DNA, 0.2 μl (50 pmol) of each primer, 5 μl of 2X Green GoTaq^® ^Reaction Buffer (pH 8.5) (GoTaq^® ^DNA Polymerase, 400 μM dATP, 400 μM dGTP, 400 μM dCTP, 400 μM dTTP and 3 mM MgCl2). The PCR amplification was carried out in a programmable thermal cycler (MJ research) using the following program: 3 minutes at 92°C, 15 seconds at 92°C, 30 seconds at 65°C, 30 seconds at 72°C followed by 39 cycles of 15 seconds at 92°C, 30 seconds at 65°C, 30 seconds at 72°C, final extension of 10 minutes at 72°C. The PCR products were size separated on 1.5% agarose gels to confirm genotype based on the amplification of target regions.

### RNA Isolation

Total RNA was isolated from left pectoralis muscle using TRIzol reagent (Invitrogen, Carlsbad, CA, USA) according to manufacturer's directions. The RNA was purified by columns (Qiagen sciences, Maryland, USA) and treated with DNase I (Qiagen, Maryland, USA) to remove genomic DNA contamination. The RNA concentration was measured by UV absorbance. RNA samples with an A_260/280 _ratio of = > 2.0 were used for RT-PCR.

### cDNA Synthesis and Real Time PCR for MYOD and Myogenin

Reverse transcription of RNA was performed (Invitrogen, Carlsbad, CA) by adding 10 μl of (500 ng) total RNA, 1 μl of Oligo (dT) 12-18 (500 μg/ml), and 1 μl 10 mM dNTP incubated at 65°C for 5 minutes and immediately chilled on ice. Then, each component was added in the indicated order 4 μl of (5×) first strand RT buffer, 0.1 M DTT 1 μl, RNaseOUT. RNase Inhibitor 1 μl, incubated at 42°C for 2 min. 1 μl (50 units) of SuperScript II RT was then added to each tube mix, and incubated at 42°C for 50 min. The reactions were terminated at 70°C for 15 min. Chill on ice.

Real-time PCR was performed on Stratagene 4000× Real-Time PCR System. Quantitative PCR reactions were optimized for annealing temperature and primer concentration. Real-time PCR reactions were performed in triplicate and template controls (NTCs) were run for each assay under the same conditions. End-point PCR was then performed in 10 μl reaction containing 5 μl SBYR green Master Mix, 3.2 μl of autoclaved nuclease free water, 1 μl diluted RT product (Minimum1:10 but MyOD undiluted cDNA was used) and 0.4 μl forward and reverse primers (specific to genes i.e MyoD F-5'-TACAGTGGCGACTCAGATGC-3' and R-5'-GCTCCACTATGCTGGACAGG-3' and MyoG F-5'-CTACAGGCCTTGCTCAGCTC-3' and R-5'-ACGATGGACGTAAGGGAGTG-3') for 40 cycles (two steps: 95°C for 10 minutes followed by 40 cycles 95°C 15s followed by 58°C for 60 s). Standard curves were generated with known amounts (molecules) of cDNA and run with each PCR run. Gene expression levels were normalized to the level of β-actin expression, which we have shown to be unresponsive to Myostatin expression level [15], and are reported relative to Myostatin wild-type expression level.

### Detection of Mature miRNAs by TaqMan assays

Five mouse Taqman miRNA assays were purchased from Applied Biosystems. Reverse transcription reactions (15 μl) containing, 1.5 μl of 10× reverse transcription buffer, 1.0 μl of MultiScribe™reverse transcriptase (50 U/μl), 0.15 μl of 100 mM dNTPs (with dTTP) and 0.19 μl of RNase inhibitor (20 U/μl), 4.16 of Nuclease-free water were mixed by brief centrifugation, 5 μl of total RNA (50 ng) was added and mixed well, 3 μl of 1× looped-primers were added (specific for microRNA) and mixed well and incubated for 30 min each, at 16°C, 42°C and 5 minutes at 85°C according to manufacturer's directions. The cDNA was diluted to a minimum of 1:15 with nuclease free water and 1.33 μl was used in real time PCR.

Real-time PCR was performed on Stratagene 4000× Real-Time PCR System. During the target amplification step, the AmpliTaq^® ^Gold DNA polymerase amplifies target cDNA synthesized from the RNA sample, using sequence-specific primers from the TaqMan Assay. Real-time PCR reactions based on TaqMan^® ^reagent chemistry were performed in triplicate and template controls were run for each assay under the same conditions. End-point PCR was then performed in 20 μl reactions that contained 10.00 μl TaqMan 2× Universal PCR Master Mix No AmpErase UNG, 7.67 μl of autoclaved nuclease free water, 1.33 μl diluted RT product (Minimum1:15) and 1 μl miRNA-specific PCR probe for 40 cycles (two steps: 95°C for 10 minutes followed by 40 cycles 95°C 15s followed by 60°C for 60 s). miRNA expression levels were normalized to the level of *miR-24 *expression, which was unresponsive to Myostatin expression level, to correct for differential cDNA content.

### Statistical analysis

Student's *t*-test was used to determine whether a significant difference existed among MSTN^-/-^, MSTN^+/- ^and MSTN^+/+ ^animals. The *P *< 0.05 was denoted by an asterisk.

## Findings

Pectoralis muscles from 29 mice were examined for differential expression of five microRNAs and two genes. The weight of MSTN^-/- ^pectoralis muscle (108 ± 5.9 mg) was significantly greater (p < 0.001) than MSTN^+/+ ^pectoralis muscle (72.6 ± 5.9 mg) 35 days after birth.

### Expression of muscle-specific miRNAs and myogenic factors

**Figure 1:**
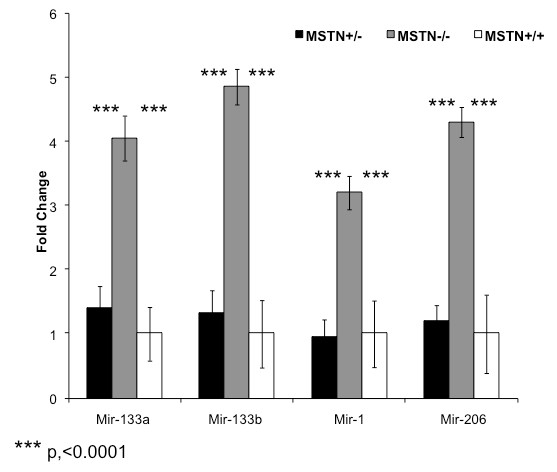
**Relative expression of mature microRNAs in the mouse pectoralis muscle**. Real-time PCR expression values were normalized to miR-24 and then reported relative to MSTN^+/+ ^levels. Data are means ± S.E. *** p, <0.0001. MSTN +/+ is a wild-type mouse, MSTN -/+ represents a heterozygous Myostatin-null mouse, and MSTN -/- represents a homozygous Myostatin-null mouse.

**Figure 2:**
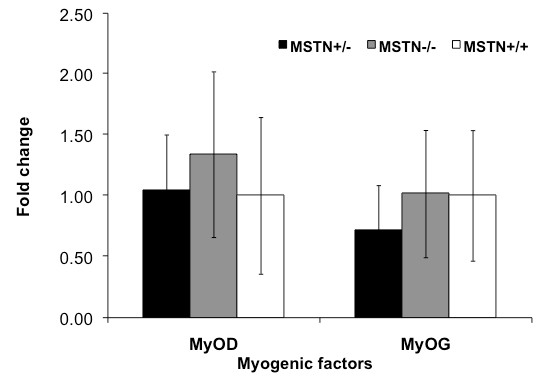
**Relative expression of myogenic factors in the mouse pectoralis muscle**. Real-time PCR expression values were normalized to β-actin and are reported relative to MSTN^+/+ ^levels. The graph represents the mean (± SE) fold change in gene expression.

The expression level of *miR-133a *(p < 0.0001), *miR-133b *(p < 0.001), *miR-1 *(p < 0.001), and *miR-206 *(p < .0001) was greater in Myostatin-null animals as compared to wild type and heterozygous animal (Figure 1). In all cases, the expression level of miRNAs was equivalent in MSTN^+/- ^and MSTN^+/+ ^muscle. In contrast, *miR-24 *expression level was not affected by Myostatin genotype (data not shown). Myostatin genotype had no significant effect on the expression level of MyoD or Myogenin (Figure 2).

## Discussion

Accumulating evidence supports a role for miRNAs in the regulation of myogenesis[16]. *miR-1/-206 *and *miR-133 *play opposing roles in modulating skeletal muscle proliferation and differentiation. The *miR-1 *and *miR-206 *promote myogenesis, while *miR-133 *inhibits myoblast differentiation and promotes proliferation by repressing serum response factor and a key splicing factor[17-20].

In this study, we observed higher *miR-1, miR-133a, miR-133b*, and *miR-206 *expression levels in the pectoralis muscle of MSTN^-/- ^mice as compared to MSTN^+/+ ^and MSTN^+/- ^animals. This increased level of *miR-1, -133a, -133b, and -206 *expressions was consistent with the enhanced skeletal muscle growth observed in MSTN^-/- ^mice. In order to sustain the increased growth observed in Myostatin-null mice elevated satellite cell proliferation, which is regulated by *miR-133*[19] and differentiation, which is regulated by *miR-1 *and *-206 *[19,19,21], must occur. However, this would also indicate that the increased levels of miRNA would need to occur in a cell-type specific manner.

Recently, Davis et al. (2008) reported that SMAD proteins control Drosha-mediated miRNA maturation. They observed that TGF-β and BMP4 treatment promoted the formation of specific miRNA/SMAD complexes, which facilitated miRNA maturation. In contrast, we observed increased mature miRNA levels in the absence of functional Myostatin, which is a TGF-β super family member. This observation indicates the possibility that TGF-β super family member can both promote and inhibit maturation of specific miRNAs[22].

Alternatively, the increased miRNA expression observed in the absence of functional Myostatin could be the result of enhanced Myf5 and MEF2A gene expression. Previously, we reported that loss of functional Myostatin increased both Myf5 and MEF2A gene expression[15]. Recently, Sweetman et al. (2008) reported that over expression of Myf5 was capable of inducing *miR-1 *and *miR-206 *[23]. While, miR-1 and miR-206 expression was lost in Myf5^-/- ^mice, which indicates that miR-1 and miR-206 lie downstream of Myf5. Similarly, Liu et al. (2007) identified an intragenic MEF2-dependent enhancer that directed miR-1 and miR-133a expression levels. Thus, it is possible that the observed increase in muscle-specific miRNAs is the result of increased Myf5 and MEF2A expression, which are the result of loss of functional Myostatin[24].

Interestingly, none of the miRNAs exhibited an additive expression level with the increasing loss of Myostatin (ie. miRNA expression in heterozygotes should be intermediate between Myostatin-null and wild-type). Myostatin appears to control *miR-1, -133a, -133b*, and *-206 *in a dominant manner, as the presence of functional Myostatin (wild-type and heterozygous mouse muscle) similarly regulated miRNA expression. Given that skeletal muscle mass was increased in heterozygous Myostatin mice, this would seem to indicate that *miR-1, -133a, -133b*, and *-206 *are not required for the observed increased muscle mass observed in Myostatin-null and heterozygous mice. Alternatively, it is also possible that *miR-1, -133a, -133b*, and *-206 *are very sensitive to Myostatin and even low levels of Myostatin, such as those observed in MSTN^+/- ^mice, can inhibit their expression. Thus, it is possible that *miR-1, -133a, -133b*, and *-206 *are necessary for the increased muscle mass observed in Myostatin-null mice, but they are not rate limiting.

## Conclusions

The expression of *miR-133a *(p < .0001), *miR-133b *(p < .001), *miR-1 *(p < .001), and *miR-206 *(p < .0001) were significantly higher in myostatin null mice compared to wild type animals. Therefore, myostatin may regulate the expression of miRNAs in skeletal muscle. Similarly, myostatin has no effect on expression of myogenic factors such as MyoD or Myogenin in the muscle tissue.

## Abbreviations

dATP: 2'-deoxyadenosine 5'-triphosphate; cCTP: 2'-deoxycytosine 5'-triphosphate; dGTP: 2'-deoxyguanosine 5'-triphosphate; dTTP: 2'-deoxythymidine 5'-triphosphate; MgCl_2_: Magnesium chloride; MSTN: Myostatin; PCR: Polymerase chain reaction; RT: reverse transcriptase; MSTN^-/-^: homozygous myostatin null mice; MSTN^+/-^: heterozygous mice at myostatin locus; MSTN^+/+^: wild type at myostatin locus

## Competing interests

The authors have no conflict of interest with any company or financial organization.

## Authors' contributions

SR contributed to, performed the breeding, genotyping of mouse, Real time PCR and Taqman assays and prepared the manuscript. YC contributed to the analysis of the data. JMR was responsible for the design of research and the manuscript. All authors read and approved the final manuscript.
